# Yokukansan promotes hippocampal neurogenesis associated with the suppression of activated microglia in Gunn rat

**DOI:** 10.1186/1742-2094-10-145

**Published:** 2013-12-05

**Authors:** Motohide Furuya, Tsuyoshi Miyaoka, Toshiko Tsumori, Kristian Liaury, Sadayuki Hashioka, Rei Wake, Keiko Tsuchie, Michiyo Fukushima, Satoko Ezoe, Jun Horiguchi

**Affiliations:** 1 Department of Psychiatry, Shimane University Faculty of Medicine, 89-1 Enya-cho, Izumo 693-8501, Japan; 2 Department of Anatomy and Morphological Neuroscience, Shimane University Faculty of Medicine, 89-1 Enya-cho, Izumo 693-8501, Japan; 3 Department of Nursing, Faculty of Health and Welfare, Prefectural University of Hiroshima, 1-1 Gakuen-cho, Mihara 723-0053, Japan; 4 Shimane University Health Administration Center Izumo, 89-1 Enya-cho, Izumo 693-8501, Japan

**Keywords:** Anti-inflammatory action, Cognitive function, Microglia, Neurogenesis, Schizophrenia (231/350), Unconjugated bilirubin, Yokukansan

## Abstract

**Background:**

The pathophysiology of schizophrenia (SCZ) remains unclear, and its treatment is far from ideal. We have previously reported that yokukansan (YKS), which is a traditional Japanese medicine, is effective as an adjunctive therapy for SCZ. However, the mechanisms underlying the action of YKS have not yet been completely elucidated. A recent meta-analysis study has shown that adjuvant anti-inflammatory drugs are effective for SCZ treatment, and it has been proposed that some of the cognitive deficits associated with inflammation may in part be related to inflammation-induced reductions in adult hippocampal neurogenesis. Although certain ingredients of YKS have potent anti-inflammatory activity, no study has determined if YKS has anti-inflammatory properties.

**Methods:**

Using the Gunn rat, which has been reported as a possible animal model of SCZ, we investigated whether YKS affects cognitive dysfunction in an object-location test and the suppression of microglial activation and neurogenesis in the hippocampus.

**Results:**

We found that YKS ameliorated spatial working memory in the Gunn rats. Furthermore, YKS inhibited microglial activation and promoted neurogenesis in the hippocampal dentate gyrus of these rats. These results suggest that the ameliorative effects of YKS on cognitive deficits may be mediated in part by the suppression of the inflammatory activation of microglia.

**Conclusions:**

These findings shed light on the possible mechanism underlying the efficacy of YKS in treating SCZ.

## Background

Schizophrenia (SCZ) is one of the most intriguing psychological disorders; it has a huge adverse impact on quality of life and requires high expenditure for treatment [1]. Although the pathophysiology of SCZ is still far from being completely elucidated, researchers have indicated hippocampal neurogenesis as a key target for the pathogenesis and treatment of this disease [2]. The hippocampus, which plays an important role in learning and memory processes and mood regulation, has been the subject of numerous studies addressing the cause of SCZ [3]. There are ample preclinical evidences suggesting that SCZ is related to decreased hippocampal neurogenesis [2]. Moreover, neuroinflammation associated with activated microglia is negatively correlated with the survival rates of hippocampal neurons [4]. A positron emission tomography-based study has shown activated microglial cells in the hippocampus of patients with SCZ during the psychotic state [5]. Moreover, previous studies have suggested that adjuvant anti-inflammatory drugs are effective for treating SCZ [6].

From the standpoint of the heterogeneity of SCZ, previous studies have indicated a close association between unconjugated bilirubin (UCB) and SCZ [7,8]. UCB activates microglia and induces the release of proinflammatory cytokines from microglia [9]. On the basis of these findings, we suggested that elevated levels of UCB play an important role in SCZ etiology, and that the Gunn rat, which exhibits a high concentration of UCB, is useful as an animal model of SCZ [10-12]. Furthermore, our previous studies have shown that Gunn rats have cognitive deficits [10] and contain activated microglia in the hippocampus [11].

We recently reported that yokukansan (YKS), which is a traditional Japanese medicine (termed 'kampo medicine’ in Japan), is effective as an adjunctive therapy for treatment-resistant SCZ [13]. To date, basic studies have suggested that both the glutamate and serotonin signaling pathways are involved in the antipsychotic action of YKS [14,15]. In addition, several *in vitro* studies have reported the neuroprotective effects of YKS [16,17] and some human studies have demonstrated the effectiveness of YKS in regulating emotion [18,19], implying that YKS promotes hippocampal neurogenesis. Tanaka and Mizoguchi demonstrated that YKS has neurogenic effects in aged rats [20]. Furthermore, facilitated hippocampal neurogenesis may be associated with the anti-inflammatory action of YKS, as several ingredients of YKS possess potential anti-inflammatory properties (Table 1).

**Table 1:** Crude drug composition of yokukansan and corresponding anti-inflammatory effects

**Crude drug name**	**Composition (g)**	**Anti-inflammatory component**
*Atractylodes lancea* rhizome	4.0	β-Eudesmol [35]
*Poria* sclerotium	4.0	Pachymic acid [37]
*Cnidium* rhizome	3.0	Senkyunolide A [30]
		Z-Ligustilide [30]
		Ferulic acid [36]
*Uncaria* hook	3.0	Rhynchophylline [32,38] Isorhynchophylline [32]
Japanese angelica root	3.0	Ferulic acid [36]
*Bupleurum* root	2.0	Saikosaponin a, c, d, e [33]
*Glycyrrhiza*	1.5	Liquiritigenin [31]
		Glycyrrhetinic acid [34]

The numbers of the corresponding references are shown in parentheses.

Recently, it was suggested that cognitive dysfunction in SCZ shows high prevalence, is relatively stable over time, and is independent of psychotic symptoms [21]. Moreover, cognitive dysfunction is present in healthy relatives of SCZ patients, and it has been suggested as a biomarker of SCZ. As a consequence, disturbances in critical cognitive process, such as spatial working memory, are regarded as a core feature of SCZ [22]. Moreover, object-location test (OLT) has been used to test cognitive dysfunction, especially spatial working memory [23].

In this study, we hypothesized that YKS is effective in treating SCZ by promoting hippocampal neurogenesis and, in part, via its anti-inflammatory actions. We investigated whether YKS affects cognitive functions involved in performing the OLT. To gain further insight into the neurobiological effects of YKS, microglial activation and neurogenesis in the hippocampus were evaluated using immunohistochemistry.

## Methods

### Animals

Seven-week-old male homozygous (j/j) Gunn rats and male Wistar rats (Japan SLC, Inc., Shizuoka, Japan) were used in this study. The rats were housed in plastic cages (39 × 27 × 18 cm) under standard conditions (temperature, 23 ± 2°C; humidity, 55 ±5 %; 12 h light/dark cycle [light phase from 07:00 to 19:00 h]) and were given free access to food and water. One week before the experiment, the rats underwent a handling procedure once daily to reduce stress during the experiment. All procedures were performed with the approval of the Shimane University Animal Ethics Committee, under the guidelines of the National Health and Medical Research Council of Japan.

### Drugs

YKS (Tsumura & Co., Tokyo, Japan) is composed of seven dried medical herbs (Table 1). Each plant material was authenticated by identifying the external morphology and marker compounds of plant specimens according to the methods of the Japanese Pharmacopoeia and the company’s standard. The seven medical herbs were mixed and extracted with purified water at 95°C for 1 h, and the extraction solution was filtered and concentrated under reduced pressure; spray-drying was used to produce dried extract powder and the extract yield was approximately 15.9%. The dosage (1 g/kg) of YKS used in this study was decided upon based on the results of previous studies demonstrating that YKS ameliorated SCZ-like symptoms [24], cognitive deficits [25], and aggressive behavior [14] in rats and mice. This dosage is approximately 20 times of the clinical dose in humans. We previously demonstrated that YKS was effective in patients with SCZ (13) and Behavioral and psychological symptoms of dementia [26]. Although the difference between an effective dosage in animals and humans is uncertain, these dosages are thought to be equivalent to ameliorate psychological symptoms. Recently, several alkaloid and triterpenoid components were detected in the plasma and brain of rats orally administered YKS [27-29]. However, pharmacokinetic studies in humans have not yet been performed. Such studies are necessary to examine the pharmacokinetics of YKS components in humans and be able to clarify the detailed mechanisms and the difference of effective dosages.

### Experimental design

The rats were divided into four groups: Wistar-control (WC) group, Wistar-YKS (WY) group, Gunn-control (GC) group, and Gunn-YKS (GY) group. The rats in the control groups (WC and GC) were given drug-free water *ad libitum* for 6 weeks, whereas those in the YKS-treated groups (WY and GY) were given water containing 0.6% YKS (corresponding to a dosage of 1 g/kg of body weight) for the same period. Each group of animals received intraperitoneal injections of 50 mg/kg bromodeoxyuridine (BrdU; Sigma-Aldrich, St. Louis, MO, USA), four times a day (with an interval of 2 h) during the last two weeks of drug dosing. The OLT for each animal was performed for 1 h after the final administration of test substance on the sixth week. After the behavioral test was finished, the rats were anesthetized deeply via intraperitoneal injection of 80 mg/kg sodium pentobarbital and perfused transcardially with 500 mL of physiological saline, followed by 500 mL of 4% paraformaldehyde in 0.1 M phosphate buffer (PB; pH 7.3). After perfusion, the brain was removed quickly and post-fixed in the same fixing solution at room temperature for 4 h. The brain was immersed overnight in 20% sucrose solution at 4°C and used later for immunohistochemical analysis.

### OLT

The OLT behavioral test was performed according to the previously described procedure [23]. The experimental apparatus used in this study was an open-field box (42 × 42 × 42 cm) made of gray polyvinyl chloride. Identical plastic columns (26 cm in height × 7 cm in diameter) were used as objects. During habituation, rats were allowed to explore the apparatus without objects freely for 1 h on the day before the acquisition trial. During the acquisition trial, each rat was allowed 5 min to explore two identical objects that were placed in the corners of the experimental apparatus. A test trial was conducted 1 h after the acquisition trial. In the test trial, one of the two objects used in the acquisition trial was placed at a new location. The rat was again placed in the open field for 5 min, and the extent of exploration was assessed by measuring the total time spent in exploratory behavior, such as sniffing, licking, or touching the object while facing it. During both the acquisition and test trials, the rat’s behavior was observed using a camera located over the open field, and recorded on videotape. All behaviors were analyzed by three blind raters. The location index (LI), which is the percentage of time spent exploring the object placed in the new location during a test trial, was calculated as follows: LI (%) = (time spent exploring the object in a new location)/(time spent exploring the object in a new location + time spent exploring the object in the familiar location) × 100.

### Immunohistochemistry

The brains were immersed in 20% sucrose solution sectioned at a thickness of 40 μm at the frontal plane using a freezing microtome. The sections were divided into four series to visualize the expression of the ionized calcium-binding adapter molecule I (Iba1, a marker of microglia/macrophages [30]), CD11b (CD11b expression is up-regulated in activated microglia [31]), Iba1/CD11b, and BrdU/NeuN. The labeling procedures for visualization of immunoperoxidase and double-immunofluorescence, and the morphometric analytical procedure were as follows.

#### Immunoperoxidase labeling

Iba1 or CD11b expression was visualized as described previously [11]. Sections treated with either rabbit anti-Iba1 (1:4000, Wako, Osaka, Japan) or mouse anti-CD11b (1:500, Serotec, Oxford, UK) antibodies were incubated overnight at room temperature on a rotatory shaker. The sections were then treated using a standard ABC Kit procedure (Vector Labs, CA, USA). Subsequently, the sections were developed by incubating in PBS containing 10 mg diaminobenzidine (DAB) and 5 μL of 30% hydrogen peroxide for 10 min.

#### Double-immunofluorescence labeling

The sections were incubated with the primary antibodies rabbit anti-Iba1 (1:4,000) and mouse anti-CD11b (1:500). Subsequently, the sections were incubated in PBS containing Cy3-conjugated anti-Iba1 rabbit IgG (1:1,000, Jackson, PA, USA) and Alexa488-conjugated anti-CD11b mouse IgG (1:1,000, Invitrogen, Oregon, USA) for 1 h at room temperature. For detecting BrdU incorporation, brain sections were incubated in 50% formamide and 2× standard sodium citrate for 2 h at 65°C, followed by incubation in 2 N HCl for 30 min at 37°C, rinsing in 100 mM boric acid (pH 8.5) for 10 min at 25°C, and washing with 0.25% Triton X-100 in Tris-buffered saline (pH 7.4). The sections were incubated with the primary antibodies (rat anti-BrdU IgG [1:10, Serotec] and mouse anti-NeuN [1:200, Millipore Inc., CA, USA]), followed by incubation with Cy3-conjugated anti-BrdU rat IgG (1:500, Abcam, Tokyo, Japan) and Alexa488-conjugated anti-NeuN mouse IgG (1:1,000, Invitrogen). All sections were visualized using a confocal laser-scanning microscope (Olympus FV-300, Tokyo, Japan) and the FluoView software (Olympus).

#### Morphometric analysis

To estimate the number of Iba1-expressing microglial cells, images were captured from different regions of the hippocampal dentate gyrus (DG): the hilus, subgranular zone (SGZ), and granular layer (GL). A 70 × 70-μm box was then placed randomly within the region of interest (from interaural 5.88 mm, Bregma -3.12 mm to interaural 4.20 mm, Bregma -4.80 mm) on the basis of a stereotaxic atlas of the rat brain [32]. For each analysis, there were about 650 boxes per region in each sample. The images were analyzed using the Stereo Investigator software Ver. 7.0 (MicroBrightField Inc., Williston, VT, USA). The number of Iba1-labeled cells in the region of interest was calculated automatically by the software. The immunoreactive area was measured using a computer-assisted image analysis program (ImageJ 1.46r; National Institutes of Health, Bethesda, MD, USA). Sections containing CD11b-labeled cells were examined under a light microscope (Nikon, ECLIPSE E800). Images (n = 20 examined at each time point) were captured from the region of interest, and the areas were analyzed using the Image J 1.46r software. The BrdU+/NeuN + double-labeled cells were counted throughout the DG (the same region as described above) by three blind raters.

### Statistical analyses

Results were analyzed by one-way analysis of variance (ANOVA) and *post hoc* Bonferroni test to determine differences among groups. Values are expressed as the mean ± SEM. Analyses were performed using the Statistical Package for the Social Sciences software. In the analyses, *P* values <0.05 were considered statistically significant.

## Results

### Effect of YKS on the performance of Gunn rats in the OLT

**Figure 1:**
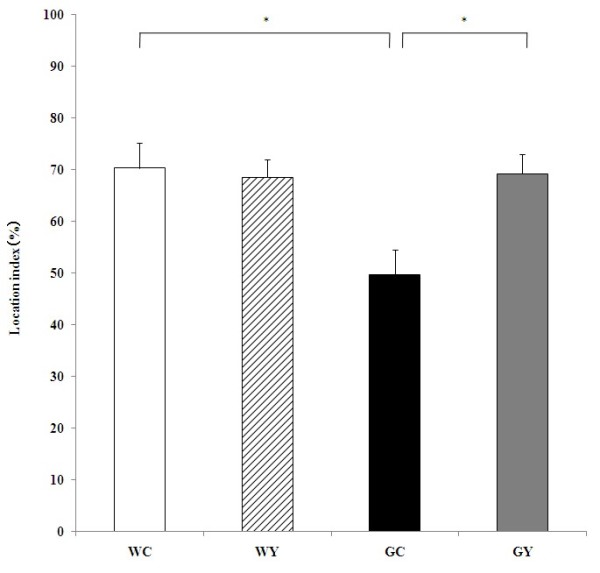
**Effect of YKS on the performance of Gunn rats in the OLT.** YKS ameliorated the cognitive dysfunction observed in Gunn rats. Data are presented as the mean ± S.E.M. (n = 4, respectively). *P <0.05, ANOVA followed by Bonferroni *post hoc* test.

Previous reports have indicated that YKS treatment stimulates neurogenesis related to cognitive function [20]. To clarify the hypothesis, we examined the effect of YKS on cognitive deficits in Gunn rats using the OLT, which is a cognitive test for spatial memory based on the spontaneous tendency of rodents to explore novel stimuli [23]. In the present study, this behavioral test was performed 6 weeks after starting YKS administration, because the critical period for enhanced synaptic plasticity in newly generated neurons is 4–6 weeks [33]. During the acquisition test, no significant differences were observed among the four groups (data not shown). On the other hand, in the test trial (Figure 1), the LI was 70.38 ± 4.85% in the WC group, 68.60 ± 3.42% in the WY group, 49.68 ± 4.94% in the GC group, and 69.28 ± 3.73% in the GY group. In other words, the effect of YKS on the LI in Wistar rats was not observed at all, but the LI in Gunn rats (GC) was significantly lower (*P* = 0.014) than that in Wistar rats (WC), suggesting that cognitive function was disturbed in Gunn rats. The lowered LI, i.e., the disturbed cognitive function in Gunn rats, was ameliorated to control level (WC) by treatment of YKS (*P* = 0.021).

### Effect of YKS on activated microglia in the DG of Gunn rats

#### Confocal microscopy of Iba1-labeled microglial cells

**Figure 2:**
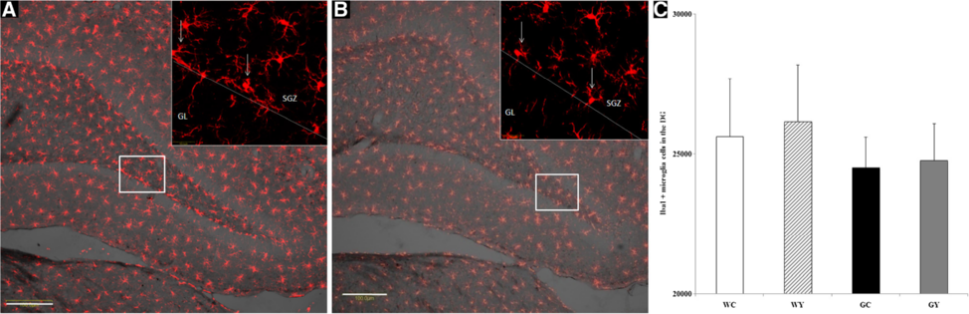
**Effect of YKS on the number of activated microglia in the DG of Gunn rats.** Microglial cells located in the SGZ of control Gunn rats (GC) **(A)** and YKS-treated Gunn rats (GY) **(B)**. There were no significant differences in the number of microglial cells in the DG among all groups **(C)**. SGZ: subgranular zone, GL: granular layer. Scale bar, 100 μm. Data are presented as the mean ± SEM (n = 4).

Microglial cells were located in the SGZ of the GC and GY rats (Figure 2A,B). There was no significant difference in the number of microglial cells in the DG among the four groups (Figure 2C): 25,623.50 ± 2067.45 in the WC group, 26,248.83 ± 2047.80 in the WY group, 24,508.83 ± 1099.36 in the GC group, and 24,771.50 ± 1314.260 in the GY group.

#### CD11b immunoreactivity in Iba1-labeled microglial cells

**Figure 3:**
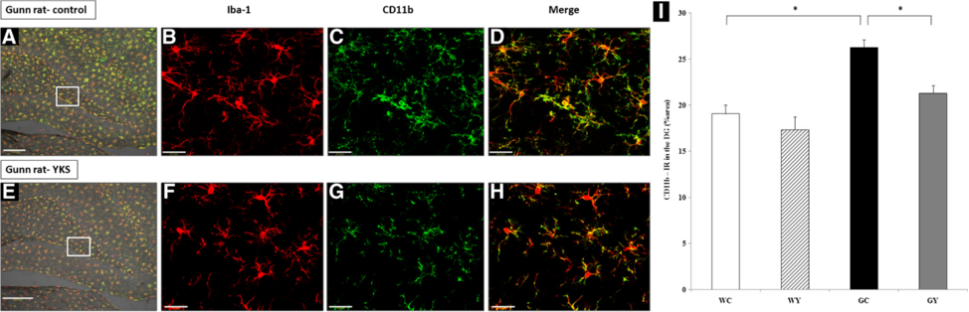
**Effect of YKS on the expression of activated microglia in the DG of Gunn rats.** YKS suppressed microglial activation in the DG of Gunn rats. A confocal Z-stack image depicting Iba-1 (red), CD11b (green), and a merged image (yellow) of cells in the DG of control Gunn rats **(A–D)** and YKS-treated Gunn rats **(E–H)**. Microglial cells in YKS-treated Gunn rats exhibited a low level of CD11b immunoreactivity (IR) compared with those in control Gunn rats **(C**, **G)**. Scale bar, 100 μm **(A**, **E)**, 50 μm **(B–D**, **F–H). (I)** Mean percentage of black pixels indicating CD11b immunoreactivity in the DG. Data are presented as the mean ± SEM (n = 4, respectively). *P <0.05, ANOVA followed by Bonferroni *post hoc* test.

Next, we evaluated the effect of YKS on microglial activation in the DG. CD11b expression in Iba1-labeled microglial cells in the DG was compared between the GY and GC groups (Figure 3A–H). Our results showed that, compared with the microglial cells in the GC group (Figure 3C,D), microglial cells in the GY group (Figure 3G,H) exhibited lower levels of CD11b immunoreactivity. Furthermore, we quantified CD11b immunoreactivity in the DG for all groups. The mean percentage of black pixels for CD11b immunoreactivity in the DG was 19.05% (% area) in the WC group, 17.33% in the WY group, 26.25% in the GC group, and 21.29% in the GY group. Statistical analysis showed that the CD11b immunoreactivity in the DG of the GY group was 18.9% lesser than that in the GC group (*P* = 0.034). The CD11b immunoreactivity in the DG was significantly higher in the GC group than in the WC group (*P* = 0.02). We observed no significant differences between the WC, WY, and GY groups (Figure 3I).

### Effect of YKS on neurogenesis in the DG of Gunn rats

**Figure 4:**
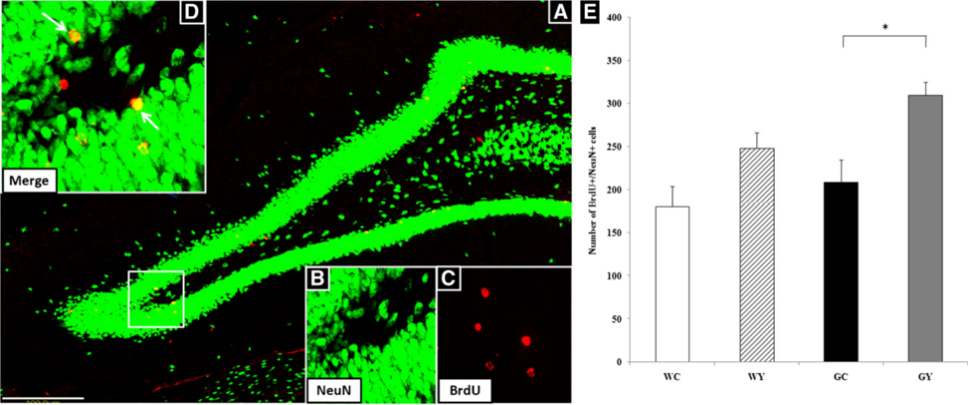
**Effect of YKS on neurogenesis in the DG of Gunn rats.** YKS increased the survival of newly born neurons in the DG. **(A)** Survival of newly generated cells in the DG. **(B–D)** Representative high-magnification photomicrograph of confocal images of the area depicted by the white square in **A** confirmed the colocalization of both BrdU and NeuN staining. **(D)** Arrows indicate double-positive cells (yellow). **(E)** YKS increased the number of BrdU+/NeuN + cells in the DG of Gunn rats. Scale bar, 100 μm **(A)**. Data are presented as the mean ± SEM (n = 4, respectively). *P <0.05, ANOVA followed by Bonferroni *post hoc* test.

To determine the survival of newly born neurons in the DG, the number of NeuN + cells among BrdU-labeled cells (BrdU+/NeuN + cells) was examined immunohistochemically 4 weeks after the final injection of BrdU (Figure 4A–D). The number of BrdU+/NeuN + neurons was 180.13 ± 24.11 in the WC group, 248.75 ± 17.38 in the WY group, 208.50 ± 26.37 in the GC group, and 309.75 ± 15.62 in the GY group. YKS (GY group) significantly ameliorated the decrease in the number of cells observed in the GC group (*P* = 0.034). There were no significant differences between the WC, WY, and GC groups (Figure 4E).

## Discussion

The purpose of the present study was to determine whether chronic YKS treatment ameliorates the cognitive deficits associated with SCZ in Gunn rats. Furthermore, we investigated whether YKS suppresses microglial activation and promotes neurogenesis in the DG of Gunn rats. This study yielded three major findings, which are discussed below.

First, YKS ameliorated the cognitive dysfunction of Gunn rats. This finding suggests that YKS enhances the integration of newly born cells into the existing neural network in the DG. In our previous study, a cognitive deficit was observed in Gunn rats during the prepulse inhibition test [10]. The perirhinal cortex [34] and hippocampus [35] are involved in object-recognition and object-location memory, respectively. Moreover, performance in the OLT relies on an intact DG, and not on other subregions [36]. Accordingly, the OLT was used in the present study to determine if YKS restores the function of the hippocampus through neurogenesis. Our result showed that amelioration of cognitive function in the OLT by YKS is consistent with the findings of a previous study that used the prepulse inhibition test [24]. To the best of our knowledge, the effects of YKS on cognitive function in the animal models of SCZ have only been demonstrated in the present study (using Gunn rats) and in a study, performed by Makinodan, using poli (I:C) [24]. However, there were no significant differences between the WC, WY, and GC groups (Figure 4E). These results suggested that the behavioral abnormalities of Gunn rats do not relate to the dysfunction in neurogenesis in the hippocampus and that YKS ameliorated the cognitive deficit in Gunn rats through a mechanism other than the increase of neurogenesis in the hippocampus.

Second, YKS suppressed microglial activation in the DG of Gunn rats. We presume that this result reflects the potent anti-inflammatory activity of YKS [37-47]. A highly characteristic feature of microglia activation is the remarkable capacity of cell populations to expand, especially in response to acute injury. This expansion is usually transient and has been considered to be predominantly due to proliferation of activated microglia cells [48,49]. Our results showed no significant difference between the numbers of microglia cells in Gunn rats compared to the controls. However, we found a significant difference in microglial activation, which was suppressed by the administration of YKS. These results suggest that microglial cells in adult Gunn rats showed a feature of activated microglial cells without expansion of the cell population, indicating chronic microglial activation. A recent meta-analysis study has suggested that anti-inflammatory drugs, such as COX-2 inhibitors, aspirin, and minocycline, are effective in SCZ treatment [6,48,49]. Kohman et al. reported that the minocycline-induced spatial learning improvement in aged mice might be related to inhibition of microglial cells [4]. They observed a significant reduction in the average the size and total number of Iba-1-positive cells in the molecular layer of the DG following minocycline treatment. However, long-term use of these drugs increases the risk of complications, i.e., COX-2 inhibitors [50], aspirin [51], and minocycline [52] induce adverse cardiovascular events, gastric ulcers and gastrointestinal bleeding, and emergence of drug-resistant strains of bacteria, respectively. Therefore, it is difficult to use these drugs in clinical treatment for a prolonged period. YKS has exhibited high safety and tolerability in several clinical studies [13,18,19]. Nevertheless, although we demonstrated that YKS suppressed the microglial activation in this study, we did not evaluate its effect on inflammatory markers, such as proinflammatory cytokines and chemokines. Therefore, further studies are needed to determine if YKS has anti-inflammatory potency.

Third, YKS promoted the survival of new neurons in the DG of Gunn rats. We suppose that this finding was, in part, due to the suppression of activated microglia in the DG by YKS. Several studies have suggested that the inflammation associated with activated microglia is detrimental to the survival of new hippocampal neurons [53]. In addition, inflammation affects functional integration of newborn neurons [54]. A study using postmortem brain material suggested that neurogenesis is more related to the pathophysiology of SCZ than to that of depression [55]. Furthermore, several neuroimaging studies have shown that hippocampal volume and activity are significantly decreased in SCZ [56]. In addition, *disrupted-in-schizophrenia 1*, which is one of the most widely investigated SCZ susceptibility genes, regulates the integration of newly generated neurons in the adult brain [57]. Therefore, we evaluated whether YKS affects hippocampal neurogenesis in the DG of Gunn rats. The mechanisms through which inflammation reduces hippocampal neurogenesis are still not fully understood, but evidence indicates that activation of microglia and the subsequent release of inflammatory molecules creates an environment that does not support survival of new cells. Evidence suggests that classic proinflammatory cytokines, IL-1b, IL-6, and TNF-α contribute to the inflammation-related decrease in neurogenesis [48,49]. Therefore, studying the effects of YKS on the production of classic proinflammatory cytokines, IL-1b, IL-6, and TNF-α is thoroughly important.

Finally, in this study, we predicted that hippocampal neurogenesis would be significantly decreased in Gunn rats compared to Wistar rats. Despite the fact that the *in vivo* study of the ameliorative effects of YKS on cognitive dysfunction is not the first to be performed, and although the evidence of cognitive recovery, microglial activation suppression, and promotion of neurogenesis by YKS shown herein are very important, we could not conclude their relation through the experiments performed in this manuscript. However, surprisingly, our results showed no significant differences in the number of surviving newly generated neurons between Gunn and Wistar rats. We speculate that this finding might represent a phenomenon of compensation by neuronal homeostasis since Gunn rats have high concentrations of UCB, which is detrimental to the CNS of neonates [58]. Furthermore, we presume that the total number of hippocampal neurons in Gunn rats decreases more than in Wistar rats.

### Limitations

First, we had investigated about the antipsychotic effects of sole YKS, unfortunately, we had no data comparing YKS with other antipsychotics; therefore, we could not mention the differences between effects of YKS and other antipsychotics. Second, in Figure 4 of the effect of YKS on neurogenesis in the DG, it seems to increase even if in DG of Wistar though it was not significant. We should demonstrate weather there was any tendency about this change, and the possible reasons why it caused especially in Gunn rats. However, it is very difficult to explain these results. However, these points are very important subjects, so that we are investigating about these subjects.

## Conclusions

To the best of our knowledge, this is the first *in vivo* study showing that YKS ameliorates cognitive dysfunction, suppresses microglial activation in the DG, and promotes hippocampal neurogenesis. The effects of YKS in SCZ may occur partly via the suppression of the inflammatory activation of microglia. Our results suggest a possible mechanism to explain the efficacy of YKS in SCZ and contribute to a better understanding of the pathophysiology of this disease.

## Abbreviations

DAB: Diaminobenzidine; DG: Dentate gyrus; GC: Gunn-control; GL: Granular layer; GY: Gunn-YKS; Iba1: Ionized calcium-binding adapter molecule I; LI: Location index; OLT: Object-location test; SCZ: Schizophrenia; SGZ: Subgranular zone; UCB: Unconjugated bilirubin; WC: Wistar-control; WY: Wistar-YKS; YKS: Yokukansan.

## Competing interests

The authors declare that they have no competing interests.

## Authors’ contributions

MF and TM contributed to conception and design of experiments; TT, MF, TM, KL, KT, MF, and SE performed immunohistochemistry, behavioral test, and data analysis and interpretation; MF, TM, TT, KL, SH, RW, and JH were involved in writing and/or critically reviewing the article. All authors have read and approved the final manuscript.
